# Low-pressure pulmonary artery aneurysm presenting with pulmonary embolism: a case series

**DOI:** 10.1186/1752-1947-5-163

**Published:** 2011-04-26

**Authors:** Eva Serasli, Μaria Antoniadou, Paschalis Steiropoulos, Konstantinos Vassiliadis, Stamatia Mantzourani, Pavlos Papoulidis, Venetia Tsara

**Affiliations:** 1 2nd Chest Department, General Hospital "G. Papanikolaou," (Exohi), Thessaloniki, GR-57010, Greece; 2 1st Department of Cardiology, General Hospital "G. Papanikolaou," (Exohi), Thessaloniki, GR-57010, Greece; 3 Department of Cardiothoracic Surgery, General Hospital "G. Papanikolaou," Exohi, Thessaloniki, GR-57010, Greece

## Abstract

**Introduction:**

Pulmonary artery aneurysm is an uncommon disorder with severe complications. The diagnosis is often difficult, since the clinical manifestations are non-specific and the treatment is controversial, as the natural history of the disease is not completely understood.

**Case presentation:**

We describe the cases of two patients with pulmonary artery aneurysms. The first patient was a 68-year-old Caucasian man with an idiopathic low-pressure pulmonary artery aneurysm together with a pulmonary embolism. The patient preferred a conservative approach and was stable at the 10-month follow-up visit after being placed on anti-coagulant treatment. The second patient was a 66-year-old Caucasian woman with a low-pressure pulmonary artery aneurysm also presented together with a pulmonary embolism. The aneurysm was secondary to pulmonary valve stenosis. She received anti-coagulants and, after stabilization, underwent percutaneous balloon valvuloplasty.

**Conclusion:**

Pulmonary embolism may be the initial presentation of a low-pressure pulmonary artery aneurysm. No underlying cause for pulmonary embolism was found in either of our patients, suggesting a causal association with low-pressure pulmonary artery aneurysm.

## Introduction

Pulmonary artery aneurysm (PAA) is a rare condition [1], and the precise incidence of the disease is unknown [2]. A true aneurysm is defined by dilation of all three layers of the vessel wall. The lesion involves the pulmonary trunk and may also extend to the main branches and the peripheral pulmonary arteries. A PAA may be an accidental finding on a chest radiograph, or it may be complicated with compression of adjacent structures, dissection, rupture or thrombus.

In some patients, PAA may be associated with significant primary or secondary pulmonary hypertension, which poses a high risk of dissection and rupture [3,4], while low-pressure PAAs seem to be more benign [5,6]. As the natural history of the disease is not well understood, the treatment is often controversial. We present the cases of two patients with low-pressure PAAs that were complicated by pulmonary embolism (PE), highlighting the diagnostic approach and the management of the patients.

## Case presentation

### Case 1

**Figure 1:**
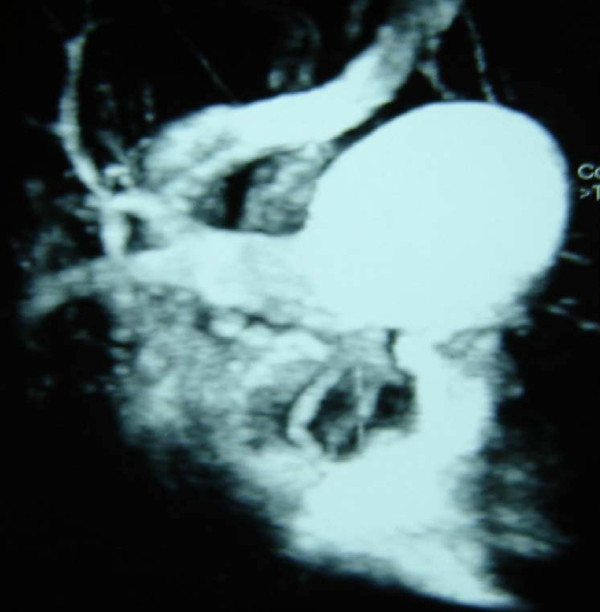
**Chest spiral CT angiography of the first patient (Case 1) showing the aneurysmal dilation involving the pulmonary trunk and its bifurcation**.

A 68-year-old Caucasian man presented to our hospital with acute shortness of breath and left-sided chest pain. He had no significant medical history. His physical examination revealed that his chest auscultation was normal and that he was normotensive. The arterial blood gas measurement showed respiratory failure with partial pressure of oxygen (pO_2_) = 55 mmHg, partial pressure of carbon dioxide (pCO_2_) = 30 mmHg, pH = 7.42 and alveolar-arterial gradient [p (A-a) O₂] = 57 mmHg on room air. His electrocardiogram revealed sinus tachycardia. The chest radiograph showed left hilar opacity. His serum D-dimer concentration was markedly elevated, and all routine laboratory tests were within normal limits. Spiral computed angiography of the chest revealed filling defects in a peripheral branch of the left pulmonary artery, suggestive of PE, and an 8.63 cm aneurysm involving the pulmonary trunk and both pulmonary arteries (Figure 1). Subcutaneous administration of low-molecular-weight heparin (LMWH) at a therapeutic dose was started, followed by oral acenocoumarol. Spiral computed angiography of the lower extremities showed no evidence of thrombi. His echocardiogram revealed normal valves, normal atrial and ventricular dimensions and normal systolic and diastolic function. The clinical and laboratory investigations were negative for infections or connective tissue diseases. The lung function tests were also within normal range. Therefore, we classified the PAA as idiopathic. Moreover, no underlying cause for the patient's PE was found. Given the large size of the aneurysm and its potential association with the thrombotic event, surgical intervention was suggested. The patient refused any invasive management, and he was discharged with normal respiratory function on acenocoumarol treatment. He was stable at the 10-month follow-up visit, when a new spiral computed tomography (CT) angiogram showed no changes in terms of the PAA dimensions, and no signs of past or newly recurrent pulmonary emboli were present.

### Case 2

**Figure 2:**
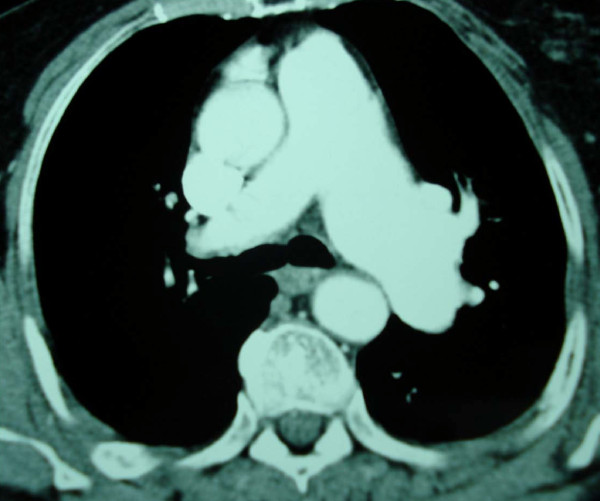
**Chest spiral CT angiography of the second patient (Case 2) reveals an aneurysmal dilation of the pulmonary trunk and the left pulmonary artery**.

Our second patient was a 66-year-old Caucasian woman who was referred to our clinic complaining of progressive worsening of dyspnea. She had a history of non-productive cough and effort-related shortness of breath for the preceding five years. Her physical examination showed that she was hemodynamically stable, with an increased breath rate of 22 breaths/minute and a left parasternal systolic murmur. The arterial blood gas examination on room air revealed pO2 = 57 mmHg, pCO2 = 32 mmHg, pH = 7.40 and p (A-a) O₂ = 53 mmHg. Chest radiography showed left hilar enlargement. Her routine laboratory tests were unremarkable. The chest computed angiography revealed PE involving a segmental branch of the left pulmonary artery and an aneurysmal dilatation of the pulmonary trunk and the left pulmonary artery, with a maximal diameter of 4.5 cm, compressing the left main bronchus (Figure 2). Spiral computed angiography of the lower extremities was normal. A therapeutic dose of LMWH was prescribed, and the clinical status of the patient gradually improved. Transesophageal echocardiography confirmed severe stenosis of a calcified pulmonary valve, with a gradient of 62 mmHg across it and moderate right ventricular enlargement. The options of surgical correction of pulmonary valve stenosis with concomitant repair of the aneurysm versus transcatheter balloon valvuloplasty were discussed. The patient preferred to undergo percutaneous balloon valvuloplasty. Cardiac catheterization from the right femoral vein was carried out, and four balloons were placed optimally through the stenotic valve. She was discharged the next day after undergoing transthoracic echocardiography confirmed reduction of the gradient across the pulmonary valve at 40 mmHg. The patient remains well under anti-coagulant therapy, and at her three-month follow-up examination the maximal diameter of the PAA was reduced to 3 cm.

## Discussion

These two cases demonstrate a rare anatomical entity with an unusual first clinical presentation as PE. Furthermore, the management of such cases requires individualization, according to the primary cause, whereas long-term clinical and radiological follow-up is necessary, taking into consideration the potentially fatal complications.

According to the literature, PAA is an unusual lesion which can be associated with congenital heart diseases, pulmonary artery hypertension, pulmonary valve stenosis, connective tissue diseases (such as Marfan syndrome) and vasculitis. Other causes include infections [such as tuberculosis (TBC), syphilis, bacteria or fungi], atherosclerosis, hypertension, hereditary hemorrhagic telangiectasia, cystic media necrosis, Hughes-Stovin syndrome and trauma [2]. It seems that intrinsic weaknesses of the arterial wall in combination with increased hemodynamic stress are responsible for its formation [3]. The clinical manifestations are non-specific, and patients may present with hemoptysis, dyspnea, chest pain, cough and evidence of left-to-right shunt. Pulmonary angiography is the gold standard for establishing the diagnosis, but new non-invasive imaging methods, such as spiral CT angiography and magnetic resonance imaging have simplified the diagnosis [7,8].

The role of surgery in PAA is controversial, and firm guidelines for the management of this disease do not exist. Surgical intervention is generally recommended to symptomatic patients and in patients with underlying diseases or complications, left-to-right shunt, pulmonary arterial hypertension and large aneurysm size [2-4,9-12]. Some authors have suggested invasive management of low-pressure PAAs when changes in right ventricular size and function resulting from pulmonary regurgitation or pulmonary stenosis are observed [5]. However, concurrent repair of the aneurysm may not be necessary, as the risk of rupture is low, but it seems to be a logical approach in cases involving open heart surgery for pulmonary valve repair. The need for close follow-up of patients with uncomplicated PAA is also emphasized [6].

In our first patient, no underlying pathology was found and the PAA was considered idiopathic, which is exceedingly rare. In the second patient, pulmonary valve stenosis and post-stenotic dilation could have been the pathophysiological basis of PAA development [11]. Given the facts that percutaneous balloon valvuloplasty is the treatment of choice for pulmonary valve stenosis [13] and that rupture of low-pressure aneurysms is rare, valvuloplasty alone appeared to be a viable management strategy.

In both patients, PAA was complicated by PE. To our knowledge, there are limited data regarding the association between low-pressure PAA and the generation of thrombi. It has been previously presumed in the literature that low-pressure PAA might be a source of recurrent emboli because of stasis and endothelial dysfunction [14]. In our patients, no other underlying cause for the thromboembolic events was found, and the causal association between PAA and PE might thus be supported. In patients without documented PE who do not undergo surgical repair of the aneurysm, the long-term use of prophylactic anti-coagulation should be evaluated. There are limited data regarding the management of this group of patients.

## Conclusion

We have presented the cases of two cases with low-pressure PAA complicated by PE. The current case report demonstrates conservative management and invasive management of two patients with idiopathic PAA and PAA secondary to pulmonary valve stenosis, respectively. As no underlying cause for PE was found in either of the patients, the embolic events seemed to be associated with low-pressure PAA. In patients with low-pressure PAA that do not respond immediately to surgical repair, further evaluation of the long-term use of prophylactic anti-coagulation is suggested.

## Consent

Written informed consent was obtained from both patients for publication of this case report and any accompanying images. Copies of the written consents are available for review by the Editor-in-Chief of this journal.

## Competing interests

The authors declare that they have no competing interests.

## Authors' contributions

ES was primarily responsible for the conception, design and revision of the manuscript. MA drafted the manuscript and searched the literature. PS was responsible for manuscript editing and advice on literature review. KV was actively involved in the patients' management and revised the manuscript. SM and PP made substantial contributions to the acquisition of data. VT approved the final version of the manuscript to be published. All authors read and approved the final manuscript.
